# Civilization and the Teeth

**Published:** 1885-02

**Authors:** 


### Civilization and the Teeth.

If the fully-evolved man of the future is to be, as has been prophesied, a hairless individual, he is only too likely to be--excepting his indebtedness to the manufacturing dentist--a toothless mortal also; for which result a persistent preference for ornament over use must be mainly held responsible.

If Helen of Troy possessed teeth as good as those of her Britannic contemporaries, she had probably as square a jaw, and a mouth of equally capable dimensions. One item in the civilized ideal of female beauty, the rose-bud mouth to-wit, is undoubtedly accountable for a great deal of the crowding and consequent injury of the teeth, especially observable in patients of the upper and middle classes, while the frequent decay of the back teeth, even before the marriageable age is reached, and the persistence of the visible front teeth till shortly after that age would seem to show that natural selection has some of the infirmities not usually associated with abstractions, and that "out of sight" is, even for it, "out of mind."

Certain of the luxuries of modern life, and the operation of some of its so-called duties, aid in completing that destructive effect against which, curiously enough, another outcome of the civilizing process—inherited gout—alone seems able to oppose its recognized attributes of large, regular, strong, and well-enameled teeth.

In the case of the negroes of the Southern States of America, a remarkable dental degeneration seems to have attended the changes in food and habits which followed the abolition of slavery. Formerly the slaves lived chiefly on corn meal and meat; at breakfast, coffee, and with dinner, vegetables were taken in addition. Occasionally wheat flour took the place of corn (maize), but it was ground on the plantation and not bolted. This food, served at regular hours, and combined with plenty of fresh air, exercise, and sleep, made the teeth strong and hard. Now the negroes eat fine wheat-flour bread, spend a large part of their wages in sweet-meats, eat at irregular times, and sleep too little.

The other side of the story is presented in a paper recently published by Dr. Kirk, who has under his care, in the Pennsylvania Institute for Deaf and Dumb, the teeth of some four hundred children. By the time that the children have been a year in this institution an entire change is noticed in the character of their teeth; they have become so hard that the instruments must be retempered in order to cut the dentine in preparing the cavities for filling; they become more firmly implanted in their sockets, and extraction is thus rendered difficult; several cases of the spontaneous arrest of carries, and of the new formation of dentine have been observed. These favorable changes are attributed to the dietary, which consists largely of various preparations rich in bone-forming materials, such as maize, oats, and wheat from which the layer just beneath the siliceous coating has not been removed in milling, together with a liberal supply of milk and a limited amount of sugar.

Another important, but only lately recognized, cause of dental decay is the undue exaction of nervous energy--probably often combined with insufficient or improper alimentation.

Recent observations have shown that carious teeth are common in modern schools in proportion to the educational standard adopted; and that the children in the higher forms have--out of all proportion to their more advanced age--worse teeth than those below them; while caries had not infrequently been noticed to commence suddenly, or to extend rapidly, during the period of examination strain. The greater work imposed upon the cerebral and other nervous centres is supposed to divert a portion of the phosphates and other mineral constituents which ought, by rights, to be devoted to the nourishment and growth of the dental structures: and it is not improbable that the secretion of the buccal glands and mucous membrane is modified under the influence of mental exertion to the deterioration of the teeth.--*Medical Times.*

